# Case of Cancer of the Penis

**Published:** 1841-10-23

**Authors:** Joseph James Ridley

**Affiliations:** Forsyth, Georgia


*Case of Cancer of the Penis.* By Joseph James Ridley, M.D.

The subject of cancer; its essential nature, its etyology, diagnosis, prognosis, &c. are involved in impenetrable obscurity; its curability remains "sub judice." The French surgeons, with one accord, acknowledge "their inability to define it satisfactorily," notwithstanding it has, since the birth of medicine, been a focus of scientific light, and intellectual giants have brought the vast resources of their minds into its exploration, it still remains a "sealed book." The very best evidence of its being "terra incognita," is the universal contrariety of opinion among medical men touching it. Some men entertain hopes of its curability. A distinguished medical gentleman, within my knowledge, insists upon it that he has often succeeded in curing cancer *emphysically*; other "nomina clara," fully assured of its incurability, make this its diagnosis. Sir E. Home, M.M. Bayle and Cayol, Messis. Cooper, Gibson, &c. regard it as alone curable by the timely and judicious use of the knife.

The etyology of cancer is a matter equally controverted. Gibson, Carmichael, and others, speak of its animalcular origin; (agreeably to their theory it is produced, as psora, by acari and other animalculæ.) Liston, Roux, Cooper, Hunter, attribute it to an hereditary predisposition, developed by adventitious causes. Bayle and Alibert cite numerous instances of its descending from parent to child. Yet we have many recorded cases of its independent, substantive existence. Adams, Wardrop, Liston have met with many instances of its origin without ostensible cause. It is not my design to canvass these different opinions, but concisely to report a case of cancer of the penis. It has no remarkable interest from novelty; it may, perchance, throw light on the pathology of lithiasis, with which it was in evident connexion.

T. D., aged 85, a soldier of the Revolution, had, until recently, been blessed with a robust, vigorous constitution,—he assured me that he had never been confined to his bed more than a day at one time throughout his long, eventful life. His mode of life was laborious,—an industrious agriculturist,—his diet was poor; his habits for many years past had been at times dissipated. His temperament was *sanguineo-nervous*: excitable and fearless. He was attacked July, 1840, with symptoms of lithiasis. Itching in prepuce, and glans penis, agreeably to Dorsey, are invariable symptoms of calculus. This person suffered acutely on this account. He had difficult and bloody micturition. These symptoms continued for some time; they at length ceased and never afterwards returned. Whether the calculus became encysted, or was dissolved by an empirical remedy, (horse radish tea) as pretended by his credulous friends, or in what manner the result was brought about, I will not conjecture. About this time a wart-like pimple was perceived about the insertion of the frenum preputii into the glans penis of the size of a pea, hard and red. Not much attention was paid to it; it remained without material alteration for five or six months, when it began to extend itself. By degrees it implicated the whole of the glans penis. Now it was that empiricism essayed its powers. Various highly inflammatory remedies were used; the effect of these was aggravation. Red precipitate had been recommended; I was consulted with regard to its propriety: I cautioned in strongest terms against thus tampering with a disease which, from the representations made of it, I was induced to believe was about to terminate in cancer. I was then requested to see and attend the case. Upon my first visit I was confirmed in my apprehensions. The diagnosis was clear and obvious. The glans penis had been nearly eroded; but a very small particle remained. The whole penis was involved in high inflammation; the cellular tissue was excessively engorged. About twelve lines from the scrotum, on the corpus spongiosum, was a tit, with everted jagged edges, through which urine dribbled in his attempts to urinate. My patient was racked continually with excruciating pain. Perhaps the surest symptom of cancer is its unintermitting pain. I anticipated at once the impracticability of effecting a cure. My only recourse was anodynes; by these the old gentleman got a little respite occasionally from the severity of his sufferings. I soon perceived that I could rely upon nothing but the knife. There were evidences that the disease had extended itself deeply into the urethra. The fitful and evanescent relief afforded by anodynes and refrigerents induced in him a delusive hope of possible cure. I frankly stated that the only possible relief was in surgery, and that even amputation would not certainly reach the root of the disease. I requested my friend Docter Roddey to see my patient with me, and consult upon the chances of cure from amputation. I went armed to operate. On the night before a copious discharge of blood from the urethra had occurred. This and other indications of a thorough implication of the whole urinary apparatus induced us to decline operating. About this time a large, ill-conditioned tumour, filled with atheromatous or cheese-like matter, appeared in each groin. Whether this was from sympathy, according to Desault and Cooper, or absorption of carcinomatous virus as in syphilitic labors, I will not presume to decide. One thing I will say, that sympathy is an unscientific term. Men are apt to throw themselves upon the doctrine of sympathy to account for phenomena difficult of solution, but which grow out of a continuous chain of physiological causes. The sufferings of the old gentleman were now so intense and unintermitting that he resolved upon amputation as a means of temporary relief. He had become so weak that it was impossible to keep him nerved long enough at any one time to make the necessary preparations, and thus he vacillated from day to day until he went down to the grave. Recently a fourth tumour, similar in character to those in the groin, made its appearance above the "*os pubis.*"

A remarkable fact connected with this case, is the perfect and uninterrupted assimilation of nutrition throughout. Liston, Cooper, &c. speak of indigestion as an invariable sequence of cancer. Although a large quantity of morphine was used, yet it did not become necessary to have recourse to aperients but very rarely during the continuance of the disease (fourteen months.) I am informed that his dejections were healthy and uniform during the whole course of his disease until the third day before his decease, at which time the powers of nature gave way, and nearly every function was suspended. The inguinal tumours continued to discharge immense quantities of matter *externally*, until within a few days of his death, when the discharge turned invariably.

The science of cancer is in its "transition state, from incertitude to demonstration." Hence the great importance to the profession of medicine, that all facts tending at all to throw light upon cancer be minutely reported, that order may spring from chaos, light from obscurity. Cancer is a disease of no ordinary magnitude, whether we regard its acuteness or suffering, its great prevalence or its direful fatality. I do not expect too much from medicine in hoping that the investigations of science may yet evolve some medicinal agent with power to stay its ravages. I know not why cancer, if undertaken in its incipiency, may not be cured. Nature contains in its vast materia medica a remedy, if timely and judiciously used, for every other human malady: why must cancer prove of necessity an "approbrium?" The results of investigation hitherto have been failure. Thus was it with variola until 1776. The profession should not abate a whit in its energy in this investigation until the cure of cancer shall have become "res adjudicate."

*Forsyth, Georgia, Oct. 14th, 1841.*

